# BENEFITS OF VOLUNTEERISM FOR RETIREES: TYPES OF VOLUNTEERING MATTER

**DOI:** 10.1093/geroni/igad104.1065

**Published:** 2023-12-21

**Authors:** Gloria Lin, Edwin Chung, Dannii Yeung

**Affiliations:** City University of Hong Kong, Hong Kong, Hong Kong; City University of Hong Kong, Hong Kong, Hong Kong; City University of Hong Kong, Hong Kong, Hong Kong

## Abstract

A recent study has disclosed that the effects of volunteering vary as a function of types of volunteering activities (Lam et al., 2021). Volunteering is one of the major social participations among retirees. Therefore, we aim to investigate which types of volunteering activities are beneficial to the well-being of retirees. A total of 719 older Hong Kong Chinese adults (Mage = 66.31, SD = 4.18, range = 60 – 80) who were retired within ten years were invited to take part in this study. Their participation in instrumental and cognitively demanding volunteering activities, life satisfaction, depressive symptoms, and cognitive functioning were assessed. In the current sample, 58.97% of the retired persons did not volunteer; and the remaining engaged in instrumental volunteering (10.15%), cognitively demanding volunteering (26.43%), or both types (4.51%). In addition, the results of ANCOVAs revealed that the retirees engaging in cognitively demanding volunteering had better cognitive functioning than those who did not volunteer at all (adj. Mdiff = .78, p = .03), while the retirees engaging in instrumental volunteering exhibited more depressive symptoms compared with those who engaged in cognitively demanding volunteering (adj. Mdiff = 2.04, p = .02) and those who did not volunteer at all (adj. Mdiff = 1.91, p = .02). These results remained significant even after controlling for sex, education, socioeconomic status, and perceived health. Our findings suggest that cognitively demanding volunteering activities should be promoted to maintain the psychological and cognitive well-being of retired persons.

